# Evaluation of Fatigue Damage Monitoring of Single-Lap Composite Adhesive Joint Using Conductivity

**DOI:** 10.3390/polym16162374

**Published:** 2024-08-22

**Authors:** Chow-Shing Shin, Shun-Hsuan Huang

**Affiliations:** Department of Mechanical Engineering, National Taiwan University, No. 1, Sec. 4, Roosevelt Road, Taipei 10617, Taiwan

**Keywords:** adhesive joint, carbon nanotubes, fatigue debonding, electrical resistance change, structural health monitoring

## Abstract

The widely used adhesive joining technique suffers from the drawback of being unable to be dismantled to examine for degradation. To counteract this weakness, several structural health monitoring (SHM) methods have been proposed to reveal the joint integrity status. Among these, doping the adhesive with carbon nanotubes to make the joint conductive and monitoring its electrical resistance change is a promising candidate as it is of relatively low cost and easy to implement. In this work, resistance change to monitor fatigue debonding of composite single-lap adhesive joints has been attempted. The debonded area, recorded with a liquid penetrant technique, related linearly to the fatigue life expended. However, it correlates with the resistance change in two different trends. Scanning electron microscopy on the fracture surface reveals that the two trends are associated with distinct failure micromechanisms. Implications of these observations on the practical use of the resistance change for SHM are discussed.

## 1. Introduction

Adhesive bonding possesses a number of advantages over conventional joining methods. It transfers load over a large area, significantly reducing stress and leading to better stiffness. There is no localized heating that may degrade the material, as in the case of weld joining. It avoids reducing the load-bearing section of the structure, introducing stress concentration, and adding extra weight, as in the case of bolt or rivet joints. For fiber-reinforced composite materials, circumventing the hole drilling helps to avoid fiber discontinuity and drilling-induced delamination. However, unlike a bolted joint, an adhesive joint cannot be dismantled to inspect for service-induced defects and degradation. Service loading conditions such as impact, occasional overload, and long-term fluctuating loading may induce joint degradation/damages that may develop into eventual structural failure.

To counteract this weakness, non-destructive evaluation (NDE) techniques have been proposed to reveal defects in adhesive joints. These include various techniques that involve acoustic waves, such as the ultrasonic pulse-echo methods [1,2,3], guided wave [4,5], phase array [6,7], acoustic microscopy [8,9], and electromagnetic acoustic transducer [10]. Non-acoustic techniques such as electromechanical impedance spectroscopy using external [11] or embedded piezoelectric sensors [12,13,14], thermography [3,15,16,17], and shearography [18,19] have also been proposed. These NDE techniques are helpful in detecting defects at the fabrication stage or late stage of failure but are relatively ineffective for bonds weakened by degradation during the service stage [20,21]. Moreover, periodic inspection using these techniques for large-scale adhesively joined structures will be very time-consuming and prohibitively expensive.

On the other hand, structural health monitoring (SHM) methods have been proposed to keep track of properties that may reflect the load-carrying capability or defect development. A number of principles have been employed for this purpose. The strain/stiffness at the joint has been monitored with back face strain gages [22,23], digital image correlation [24,25], and optical fibers with distributed [26,27] or discrete [28,29] sensors. Local strain perturbation by the initiation and development of internal defects may be monitored by the change in the full spectral shape of fiber Bragg gratings [30,31,32,33]. Acoustic emission offers another possibility for detecting the occurrence and growth of bond defects [15,34]. Perturbation in electrical conductivity/impedance has also been proposed as a means to monitor the integrity of adhesive joints. Among these techniques, conventional strain gages are prone to induce delamination if embedded inside a joint. When applied to the exterior, they disrupt an otherwise smooth surface and are susceptible to environmental degradation. Optical fibers have excellent fatigue endurance [35] and can be embedded inside a joint without causing adverse effects on the structural integrity. However, the sensing region is limited to a close vicinity of the fiber sensor. Moreover, the equipment involved in fiber sensing and acoustic emission is relatively expensive. Conductivity measurements offer a low-cost alternative that can surveil a relatively large region.

The use of electrical properties to monitor the integrity of a composite has been around for some time [36,37,38,39,40,41,42]. Early works made use of conductive carbon fiber [36] or CNT-coated glass fiber [37,38]. Conductive nanoparticles have also been dispersed in resin for this purpose [39,40]. The most popular conductive particle employed is carbon nanotubes, while graphene [41] and carbon black [42] have also been used. The latter approach has also been employed for adhesive joint monitoring. After doping with conductive particles, the adhesive was either made into a thin adhesive film [24,43,44,45,46] or applied directly [15,47,48,49,50,51,52] to fabricate the joint. Conductive sensors such as bucky paper [53], aligned carbon nanotube web [54], inkjet-printed silver nanoparticle interdigital sensors [55], and metal-pinned hybrid composite–titanium joints have also been investigated. The percentage change in D.C. resistance is normally measured in these works, but a few studies used A.C. to measure impedance [48,52]. The above works showed clearly that electrical resistance or impedance changes are associated with straining as well as the initiation and growth of a crack or debonding. The latter fact offers good potential for SHM using conductivity/resistance.

However, the considerable amount of work in this area still needs to be improved when the practical application of the technique is intended. Firstly, current results are qualitative and of a proof-of-concept nature. The practical application of this technique requires more concrete quantitative relations. Secondly, most of the current works demonstrated the phenomenon on a very limited number of specimens, and the reproducibility of results has not been thoroughly investigated. Thirdly, most of the works measured resistance under an increasing straining deformation. Resistance changes in a CNT-doped adhesive joint may result from one of the following mechanisms: (1) change in the amount of direct contact of the overlapping nanotubes [56,57]; (2) change in the tunneling resistance resulting from changes in inter-tube distance between non-touching neighboring CNTs [57,58,59]; (3) the intrinsic piezoresistivity of individual CNTs due to strain-dependent energy band gap opening [60,61]; (4) reduction in conductive cross-sectional area due to cracking and debonding growth. The first three mechanisms constitute the piezoresistive effects of the adhesive and will operate under straining without the existence of damage. Internal damage such as microcracking that leads to geometrical changes in the CNT network and residual stress redistribution can also operate the first three mechanisms. Debonding between the adhesive and adherend operates the fourth. When resistance measurement is made under load, both the contributions from straining and possible damages occur. For practical SHM applications, the isolation of the contribution from damages to reflect only structural degradation is needed.

The current work looks into the possibility of establishing a quantitative relationship between resistance change and debonding crack growth under cyclic loading. To understand the reproducibility of results, fatigue tests have been repeated on 30 specimens. Resistance changes are measured under load-free or a very small load condition to alleviate any piezoresistive effect. It was found that resistance change does not form a unique relationship with the debonded area. Fractographic evidence offered a reasonable explanation for this observation.

## 2. Materials and Methods

This investigation involved the preparation of conductive adhesive single-lap-joint specimens. The specimens were fatigue-tested while their change in conductivity was monitored. The amount of debonding at some point in these tests was also recorded using a fluorescent penetrant. Finally, the fractured surfaces are examined with a scanning electron microscope (SEM) to reveal the micromechanisms of failure and to provide more understanding of the relationship between conductivity change, debonded area, and fatigue life consumption. Experimental details about the key steps are given in the following sections.

### 2.1. Lap-Joint Specimen Preparation

**Figure 1:**
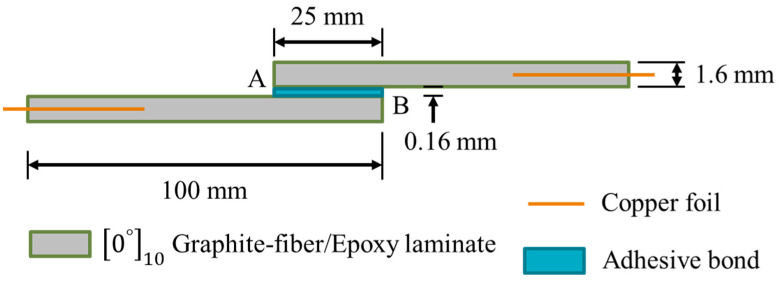
Dimensions and layout of the single-lap-joint specimen, A and B are the two longitudinal edges of the joint.

The 210 mm × 210 mm [0°]_10_ uni-directional graphite-fiber/epoxy laminates were fabricated using an autoclave vacuum bag technique at 145 °C and 10 kg/cm^2^. The graphite-fiber/epoxy prepreg (Formosa Taffeta Co., Ltd., ECU431, Douliu, Taiwan) employed has a fiber volume fraction of 63%. Copper foils were pre-embedded in the mid-layer at both ends of the laminate to facilitate later conductivity measurement. From the laminates, 25.4 mm wide strips were cut to produce single-lap-joint specimens with dimensions shown in Figure 1. A batch of seven single-lap-joint specimens can be made from each composite laminate. Adhesive joint preparation followed the procedures outlined in the ASTM D5868-01 [62].

The area to be joined was sandblasted with carborundum (CES012, Phasic Corp., Yuanlin, Taiwan), which had a mean diameter of 106–125 μm. Three 125 μm diameter optical fibers running along the specimen loading axis were embedded as spacers to control the bond line thickness to ~160 μm. The fibers were evenly spaced across the specimen width. Masking tape was applied in the immediate vicinity beyond the boundary of the joint area to prevent excess glue from forming additional but unpredictable adhesion between the two adherends.

Room-temperature-cured epoxy resin (Swancor 2261-A/BS, Swancor, Industrial Corp., Nantou, Taiwan) was made conductive by blending 0.3 wt% of amino-functionalized multi-walled carbon nanotubes (CNTs) into the resin. The manufacturer-provided specifications of the raw resin are listed in Table S1 in the Supplementary Materials. The CNTs (MWCNT-NH3-STD, Euflex Technology Corp., New Taipei City, Taiwan) have a nominal diameter of 9.5 nm and a nominal length of 1500 nm. To ensure proper dispersion, the mixture underwent sonication using an ultrasonic homogenizer (UP200S, Hielscher Ultrasonics, Teltow, Germany) at 100 W for 5 min. It was further homogenized with a high-speed homogenizer (MiniBatch D-9, MICCRA, Heitersheim, Germany) at 21,000 rpm for 10 min. The mixture was then cooled in iced water while the hardener was added and mixed. The resulting mixture, still buffered in iced water, underwent degassing in a vacuum for one hour before application. The adhesive joints were left to cure at room temperature for 24 h. Afterwards, the specimens were post-cured for 2 h at 100 °C in an oven (DOS45, Deng-Yng Corp., New Taipei City, Taiwan).

### 2.2. Conductivity Measurement

Lead wires were soldered to the exposed copper foils, which were partly embedded in the composite laminate at both ends of the single-lap-joint specimens. A constant current from a source meter (Keithley 2450, Tektronix, Inc., Beaverton, OR, USA) was applied across the specimens through the lead wires. A current of 5 mA resulted in an initial voltage drop of ~3 V, suggesting that the initial resistance of the specimen from one end to the other was ~600 Ω. A 175 mm long composite strip of the same width has a resistance of ~3 Ω. Thus, the adhesive is the primary resistance contributor to the lap-joint specimen. Changes in the corresponding voltage drop were recorded, which reflected changes in the adhesive joint resistance. Preliminary tests showed that the voltage drop, or resistance, across the joint increases with increasing load. To reflect the contribution of damage and exclude the effect of load, the specimens were brought to 500 N and 0 N every time when the voltage drop was to be recorded.

### 2.3. Mechanical Testing

The specimens underwent either tensile or cyclic fatigue loading using a servo-hydraulic testing machine (810 Materials Testing System, MTS Systems, Eden Prairie, MN, USA).

From each batch of seven lap-joint specimens, two specimens were tested under tensile monotonic loading to failure to obtain the average batch tensile strength. Different specimen batches had average batch strengths from 7.254 to 7.986 kN. However, preliminary tests showed that within the same batch, the worst-case standard deviation of tensile strength was within 4.75%.

Sinusoidal cyclic loading between 5.5% and 55% of the average batch tensile strength at 5 Hz was employed for fatigue testing. The voltage drop across the specimens was monitored throughout testing. A total of 30 specimens were tested, with an average fatigue life of 26,651 cycles and a standard deviation of 11,310 cycles. Inherently fatigue failure is a stochastic event with considerable scatter. Treatment with a liquid penetrant at different stages of life may also introduce some differences.

### 2.4. Liquid Fluorescent Penetrant Treatment

For some of the specimens, the fatigue test was interrupted, and the specimens were infiltrated with a fluorescent penetrant (Metl-Chek FP-923, McGean, Cleveland, OH, USA). The purpose of this treatment is to mark the instantaneous state of joint debonding. The treatment included immersing the specimens into the penetrant for 10 min under 0 N, followed by 20 min under 50 N. Afterward, the specimen surface was wiped dry and clean using a penetrant remover (Met-l-Chek E-59, McGean, USA). Subsequently, the specimens were baked for 48 h at 100 °C in the same oven used for post-curing to dry any entrapped penetrant thoroughly.

Fatigue testing was then resumed until specimen fracture. The fracture surface was photographed under ultraviolet (UV) illumination. The resulting images were analyzed using ImageJ 1.54J [63] to compute the fluorescent area.

### 2.5. Fractographic Observation

Representative joint fracture surfaces were gold-coated and examined using a scanning electron microscope (TM3000 tabletop SEM, Technologies Corp., Hitachi, Tokyo, Japan) to evaluate the microscopic failure mechanisms.

## 3. Results

### 3.1. Voltage Drop across the Adhesive Joint during Fatigue Testing

The percentage voltage change is defined as the following:

(1) $$\%\;voltage\;change=\frac{V-V_{0}}{V_{0}}$$

where *V*_0_ is the initial voltage drop at zero load, and *V* is the voltage drop recorded at different cycles during fatigue testing. As voltage was measured under a constant supply current, this change is equivalent to percentage resistance change.

**Figure 2:**
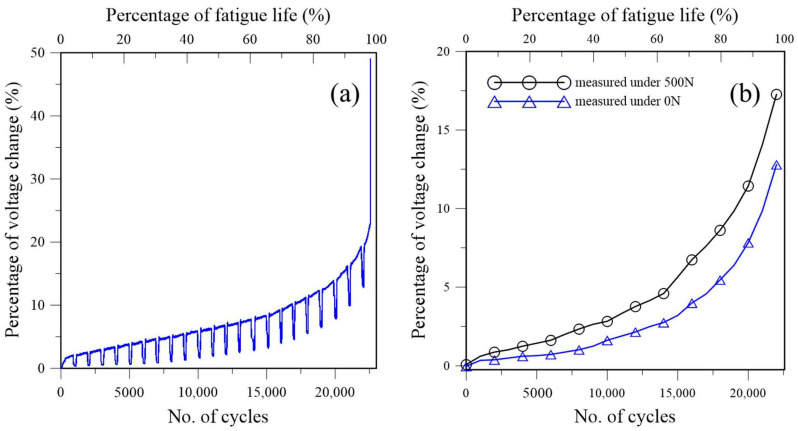
Development of the percentage voltage change in a typical fatigue test: (**a**) logged during the whole fatigue loading process; (**b**) measured at 0 N and 500 N.

Figure 2 shows the development of the percentage voltage change in a typical fatigue test without the penetrant treatment. The voltage drop continuously increases with the loading cycle. Figure 2a shows the voltage changes logged throughout the fatigue test. It is monotonically increasing with the loading cycle. Initially, the increase is very gradual. Towards the end of fatigue life, it increased steeply. Note that this voltage change, or the resistance change it reflects, contains the contributions from the piezoresistive effect due to the applied load as well as fatigue damages. The periodic dips in Figure 2a are temporary test interruptions to allow voltages to be measured at 0 N and 500 N. The latter results are presented in Figure 2b. When measured at 0 N, this increase can only be attributed to the occurrence of fatigue damage, such as microcracking and debonding in the adhesive joint. Measuring the voltage drop at 500 N may include some conductivity change due to the load, but the data before fatigue cycling in Figure 2b show that this was minimal initially (0.08%). The 500 N data are consistently higher than the 0 N data as the 500 N load tends to open up any defect resulting from the fatigue damage and increase the resistance. This effect is expected to be more marked towards the end of fatigue life, where damage is more extensive and is in fact borne from the increasingly prominent difference between the two curves as the specimen was approaching final failure.

Figure 2 suggests that through suitable calibration, the fatigue life expended, or fatigue damage incurred, may be quantified through the percentage voltage change. However, a prerequisite for this is that the relationship between voltage change and fatigue damage accumulation must be reproducible across different specimens.

### 3.2. Relationship between Fatigue Life, Debonded Area, and Percentage Voltage Change

A total of 22 specimens from five batches were successfully penetrant-treated to record the instantaneous debonded area. For these specimens, tests were interrupted when the 0 N percentage voltage change reached or just passed the targeted amounts of either 2%, 10%, or ~18%. The respective number of cycles was noted. It should be pointed out that only the debonded area that has an opening to the exterior can be recorded. The stress analysis of the single-lap joint [64] indicated that for each of the stress components, stress concentration occurs right at or very close to the longitudinal edges of the joint (positions corresponding to points A and B in Figure 1). Previous work [65] also showed that fiber Bragg grating sensors along the loading direction straddling the longitudinal joint edge are more sensitive than other configurations in detecting joint damage. These exterior edges will therefore be the most likely locations for the initiation of debonding.

**Figure 3:**
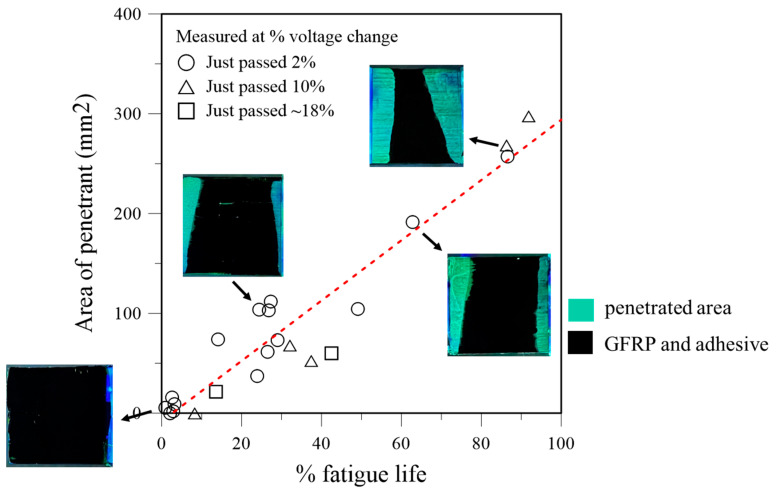
Debonded area versus percentage fatigue life expended for 22 specimens (the red dashed line represents the linear fit to the data).

The computed debonded area and the corresponding fractional fatigue life expended for each penetrant-treated specimen are presented in Figure 3; some typical photographs of the fractured joints taken under UV illumination are also shown as insets in Figure 3. Within experimental scatters, the data points lie remarkedly close to the fitted straight line, indicating that the debonded area is roughly directly proportional to the fatigue life expended. A debonded area of 200 mm^2^ roughly corresponds to 70% expended life.

Although the interruption for penetrant treatment aimed at the target percentage voltage changes of 2%, 10%, or ~18%, periodic measurement per the 1000-cycle increment often led to missing and going beyond the exact target. In fact, for the 18% aim, fatigue lives were close to the end, and several specimens simply fractured before interruption for penetrant treatment could be applied in time. This is the reason why the number of specimens for this category is limited, and one of them was treated when the percentage voltage drop reached 17.6%.

**Figure 4:**
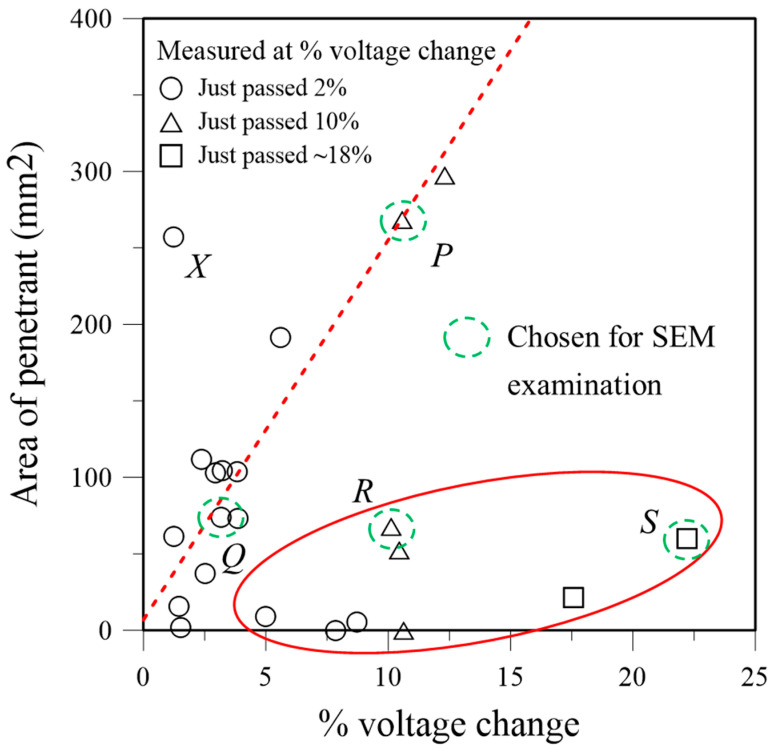
Measured percentage voltage change versus penetrant-marked debonded area for 22 specimens (the red dashed line and ellipse represent two different trends in the data).

Figure 4 plots the actually measured percentage voltage change with the penetrant-marked debond area for the specimens. Two distinct trends can be seen in Figure 4. The first involves data points falling close to the dotted line, exhibiting a linear correlation between the percentage voltage change and debonded area. The second involves data points encircled in the red ellipse that display considerable voltage changes with small debonded areas. These two distinct sets of data suggest that different failure mechanisms might prevail. A data point labeled *X* in Figure 4 falls markedly outside these two sets of data. The non-unique relation between percentage voltage change and debonded area precluded the use of the former as a quantifying parameter to describe the latter. This further implies that percentage voltage change cannot be used to successfully quantify fatigue life expended, which has a one-to-one correspondence with the debonded area. The possible causes of the non-unique relation between the percentage voltage change and the debonded area will be further elucidated through detailed fractographic examination using an SEM.

### 3.3. Fractographic Observation of Damages in the Specimens

Specimens selected from the above two data sets, together with the specimen labeled *X*, were examined using an SEM. The chosen specimens are indicated with broken circles in Figure 4. For each specimen, the fluorescent area was thoroughly scanned using magnifications from 100× to 1000× to identify characteristic features that generally prevail on the whole area. Microscopic views of these characteristic features were recorded at a number of points for each specimen. Typical views are shown in the following discussion, and more detailed micrographs are presented in the Supplementary Materials.

**Figure 5:**
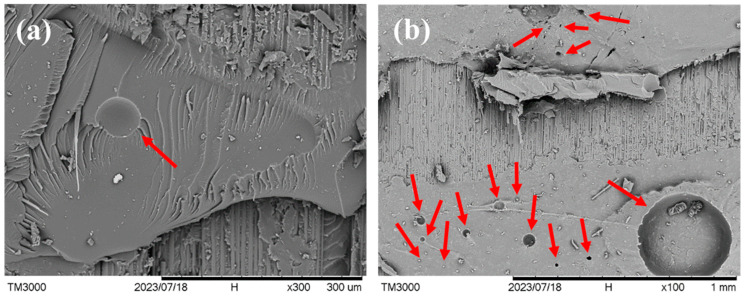
(**a**) and (**b**) Two positions on the joint adhesive of the fractured specimen *X* showing extensive gas pores (pointed to with red arrows).

Figure 5 shows magnified views of the adhesive on the joint fracture surface of specimen *X*, which has considerable debonding, but the percentage voltage change remained low. Extensive circular holes (examples of some of these are indicated by red arrows) of different sizes are evident in the adhesive. Judging from their roundness, these holes are probably residual gas pores. The presence of these gas pores reduced the cross-sectional area that is available for electric conduction. When debonding intercepts these pores, the specimen resistance will not further increase. This may explain the small change in the voltage drop even though considerable debonding has occurred.

**Figure 6:**
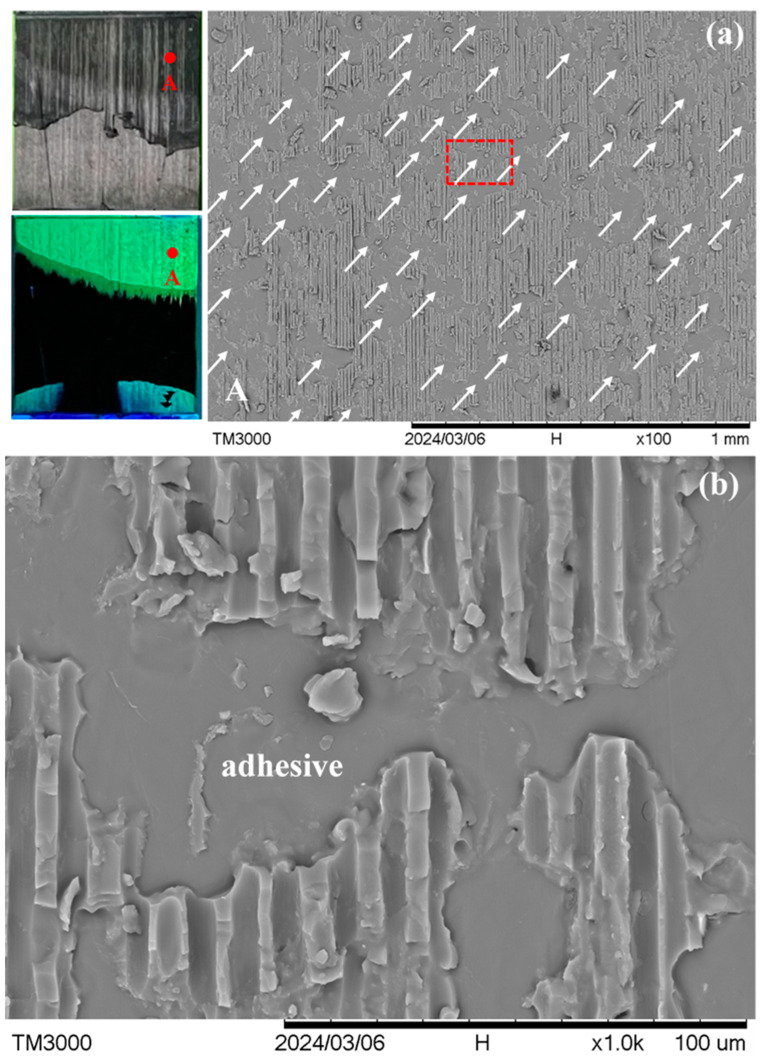
(**a**) A typical point A in visible and UV light views on the fractured joint in specimen *P* under low magnification, and white arrows show interfacial debonding sites; and (**b**) the magnified view of the dotted rectangle area in (**a**).

**Figure 7:**
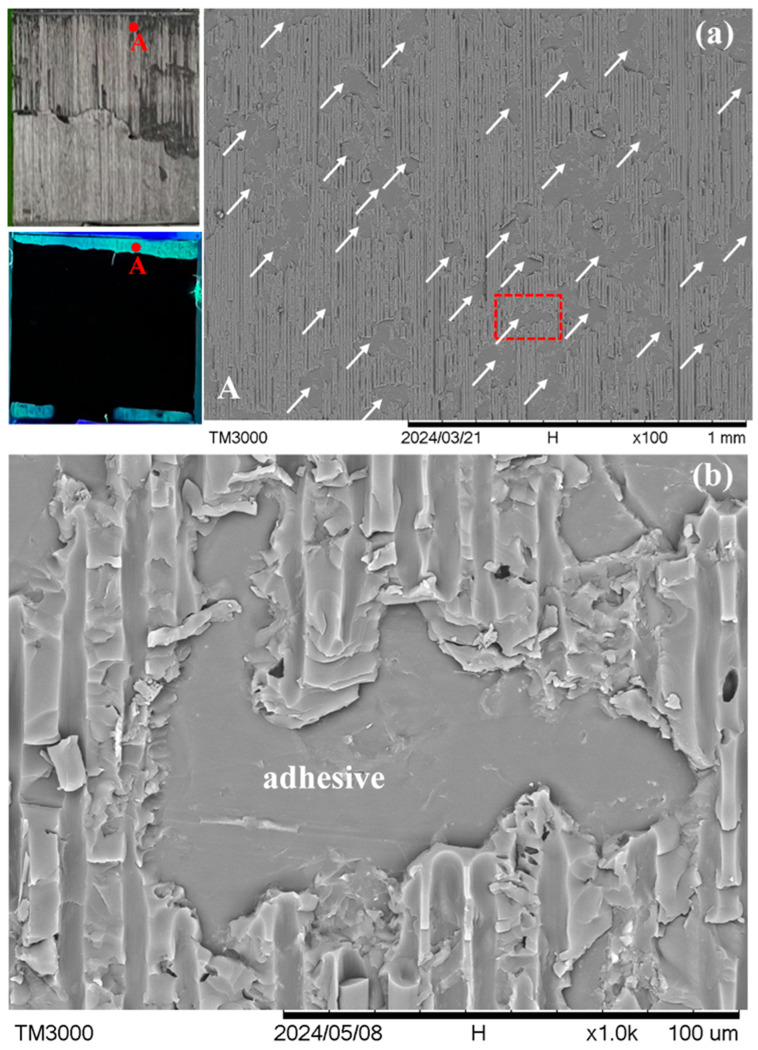
(**a**) A typical point A in visible and UV light views on the fractured joint in specimen *Q* under low magnification, and white arrows show interfacial debonding sites; and (**b**) a magnified view of the dotted rectangle area in (**a**).

Specimens *P* and *Q*, both lying on the dotted line in Figure 4, exhibited the same characteristic feature, which prevails over the whole fluorescent area. Figure 6 shows one typical point in specimen *P*. The location of this point is indicated in the macroscopic visible light and UV light views in the leftmost section of Figure 6a. The visible light view reveals the final fracture state of the entire joint. The green regions in the UV light view represent the fluorescent penetrant-soaked area. White arrows in the 100× micrograph in Figure 6a show the abundance of the characteristic feature, which consists of a relatively smooth area surrounded by rough areas with channel marks. One example of this feature, enclosed in the dotted rectangle, is magnified and displayed in Figure 6b. The channel marks have a width of ~7 μm. This dimension agrees with the graphite fiber diameter and was left by peeled-off fibers from the graphite/epoxy composite adherend. The smooth area shows no clear evidence of fiber imprints, suggesting that it is the adhesive for bonding the joint. Thus, the dominant fracture mechanisms involve partly debonding at the adhesive/adherend interface and partly the peel-off of fiber from the outermost lamina of the composite adherend. Typical fracture characteristics of specimen *Q* are shown in Figure 7. It shows the same interfacial debonding and outermost lamina fiber peel-off mechanisms. This feature also occurs in great abundance, as is evident by the arrows in the 100× micrograph in Figure 7a. More fractographs at different points on specimens *P* and *Q* are shown in Figures S1 and S2, respectively, in the Supplementary Materials.

**Figure 8:**
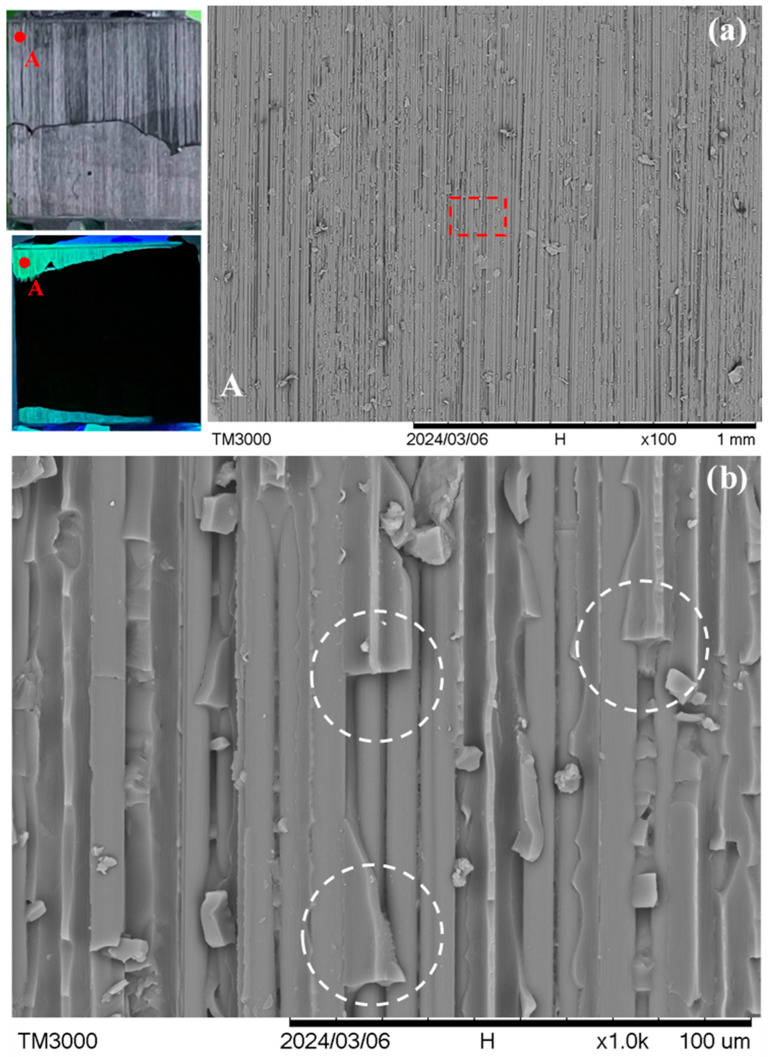
(**a**) A typical point A in visible and UV light views on the fractured joint in specimen *R* under low magnification; and (**b**) a magnified view of the dotted rectangle area in (**a**). Broken circles indicate intra- or inter-laminar debonding.

**Figure 9:**
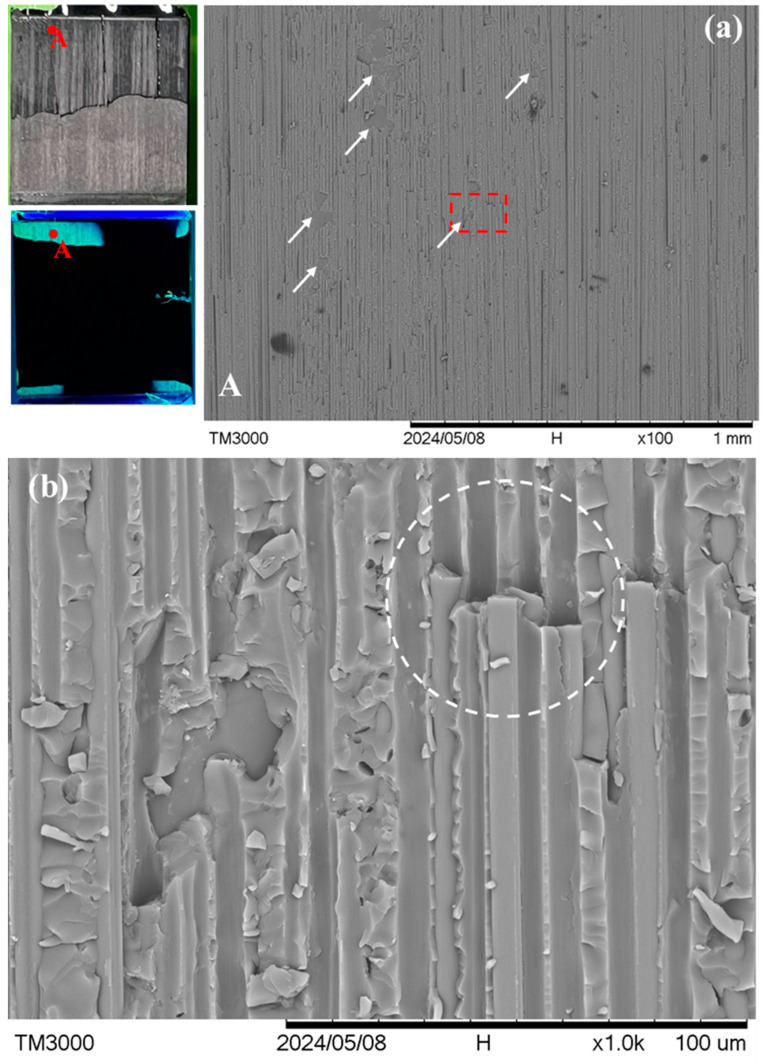
(**a**) A typical point A in visible and UV light views on the fractured joint in specimen *S* under low magnification, and white arrows show interfacial debonding sites; and (**b**) a magnified view of the dotted rectangle area in (**a**). Broken circles indicate intra- or inter-laminar debonding.

A different fracture appearance is exhibited by specimens *R* and *S*, which lie in the ellipse in Figure 4. Figure 8 shows the typical fracture appearance from one point in specimen *R*. The location of this point is indicated in the macroscopic visible light and UV light views in the leftmost section of Figure 8a. In the 100× micrograph in Figure 8a, interfacial debonding between the adhesive and adherend is rarely seen across the whole fluorescent area. The fracture surface mainly consists of fiber peel-off. Moreover, from the magnified view in Figure 8b, fibers can be seen beneath the channels of this peel-off layer (see the circled regions for examples). This indicates that debonding occurred between two graphite/epoxy laminae or inside a lamina in the composite adherends. Specimen *S* shows occasional adhesive/adherend interfacial debonding. However, its occurrence (indicated by arrows in the 100× micrograph of Figure 9a) is much less extensive than that in specimens *P* and *Q*. Again, the circled region in the magnified view in Figure 9b suggests intra- or inter-laminar debonding (broken circle) instead of interfacial debonding between the adhesive and the adherend. More fractographs at different points on specimens *R* and *S* are shown in Figures S3 and S4, respectively, in the Supplementary Materials.

The lap-joint specimen is conductive because the adherends and the adhesive are both conductive. The adhesive is conductive as carbon nanotubes have been dispersed in the resin. Although the epoxy resin in the composite adherend is non-conducting, the high volume fraction of graphite fibers is packed very closely together and inevitably, there are direct contact and tunneling points along the fiber length. In fact, contact between fibers was encouraged by the compressive stress of ~10 atmospheric pressure that was maintained throughout the whole course of composite laminate fabrication. This would press the fibers tightly together and squeeze out part of the resin between the fibers when the former was still flowable. As a result, the composite adherend is conductive and in fact has a very low resistance as mentioned before. Sandblasting the surface to be joined lightly removed some of the resin and exposed the fiber in the outmost layer at some points. At these sites, the fibers can come into contact with, or very close to, the carbon nanotubes in the adhesive, completing the conductive pathway across the specimen. From the extensive interfacial debonding revealed in Figure 6 and Figure 7, the debonded interface areas are relatively smooth and free from fiber imprint marking. This suggests that the contact between the exposed fibers and the adhesive is probably not very extensive nor tight at the joint region. A large fraction of the interfacial area may therefore have played little role in electrical conduction. As a result, intra- or inter-laminar debonding in the adherend will cause a more prominent resistance or voltage drop increase than interfacial debonding between the adhesive and adherend. This explains why the specimens inside the ellipse in Figure 4 give a significant increase in the voltage drop with a small amount of debonded area, as specimens in this category mainly exhibited intra- or inter-laminar debonding, as shown in Figure 8 and Figure 9. On the other hand, specimens close to the dotted line in Figure 4 exhibited extensive interfacial debonding between the adhesive and adherend. As a large fraction of the interface may not be involved in electrical conduction, their debonding will not affect the resistance. In this case, much more debonded area is required to induce a significant increase in the resistance or voltage drop. The prevalence of two different micromechanisms in different specimens may probably be attributed to comparable adhesive/adherend interfacial strength and the inter- or intra-laminar debonding strength.

The current study demonstrated that when competing micromechanisms are involved in the failure of an adhesive joint, the resistance and the degree of joint degradation are not uniquely correlated. This is unfavorable if conductivity is intended to be used for SHM in practical applications. The failure of an adhesive joint may involve cohesive failures in the adhesive or in any one of the adherends, as well as adhesive failure at the adhesive/adherend interface. When any two or more of these failure modes are of comparable strengths, non-uniqueness of the resistance and damage status will probably occur, as exemplified in this work. Thus, a sufficient number of specimens tested under correctly simulated service loading conditions and environment is needed in order to establish a correlation between resistance and structural integrity for use in practical SHM.

The above observation may be further tested by using the resistance change to monitor fatigue damage progression when a single micromechanism predominates. A possible material system is the metal–metal adhesive joint.

## 4. Conclusions

Attempts have been made to monitor the fatigue damage of single-lap-joint composite specimens using conductivity change and debonded area. Conductivity change is reflected by the percentage voltage change when a constant current passes through the specimen. An instantaneous debonded area was recorded with a fluorescent liquid penetrant treatment. It was found that

1. The percentage voltage change monotonically increased from 0% to 49% with fatigue loading cycles. The percentage voltage changes measured at 500 N are higher than that at 0 N, with the difference initially at 0.11% and increasing to 4.48% as it approached the end of the fatigue life.
2. The debonded area is roughly directly proportional to the fatigue life expended. A debonded area of 200 mm^2^, out of the total joint area of 625 mm^2^, roughly corresponds to 70% expended life.
3. The percentage voltage change does not have a unique correlation with the debonded area. Two different trends existed. In one trend, the percentage voltage change correlated linearly with the debonded area. Specimens in this trend are fractured partly by interfacial debonding and partly by the peel-off of fiber from the outermost lamina of the composite adherend. In another trend, considerable voltage changes occurred with small debonded areas. The major fatigue fracture mechanism for specimens in this latter trend is intra- or inter-laminar debonding inside the adherend.
4. As there is no unique correlation between the debonded area and the percentage voltage change, the latter is not a valid quantifying parameter for the fatigue life expended in the current composite lap-joint specimens.

## Data Availability

The original contributions presented in the study are included in the article/Supplementary Materials; further inquiries can be directed to the corresponding author.

## Supplementary Materials

The following supporting information can be downloaded at: https://www.mdpi.com/article/10.3390/polym16162374/s1, Table S1 The manufacturer provided specifications of Swancor 2261-A/BS epoxy resin (Swancor Industrial Corp., Nantou, Taiwan). Figure S1. More microscopic and magnified views of the dotted rectangle joint fracture views of specimen *P*; extensive white arrows show interfacial debonding sites. Figure S2: More microscopic and magnified views of the dotted rectangle joint fracture views of specimen *Q*; extensive white arrows show interfacial debonding sites. Figure S3: More microscopic and magnified views of the dotted rectangle joint fracture views of specimen *R*; sparing white arrows show interfacial debonding sites and broken circles indicate intra- or inter-laminar debonding. Figure S4: More microscopic and magnified views of the dotted rectangle joint fracture views of specimen *S*; sparing white arrows show interfacial debonding sites and broken circles indicate intra- or inter-laminar debonding. Refs. [66,67,68] are listed in References of main text.

## References

[1] Casavola C., Palano F., De Cillis F., Tati A., Terzi R., Luprano V. (2018). Analysis of CFRP Joints by Means of T-Pull Mechanical Test and Ultrasonic Defects Detection. Materials.

[2] Jasiuniene E., Mazeika L., Samaitis V., Cicenas V., Mattsson D. (2019). Ultrasonic non-destructive testing of complex titanium/carbon fibre composite joints. Ultrasonics.

[3] Yilmaz B., Ba A., Jasiūnienė E., Bui H.-K., Berthiau G. (2020). Evaluation of Bonding Quality with Advanced Nondestructive Testing (NDT) and Data Fusion. Sensors.

[4] Yu X., Fan Z., Puliyakote S., Castaings M. (2018). Remote monitoring of bond line defects between a composite panel and a stiffener using distributed piezoelectric sensors. Smart Mater. Struct..

[5] Ochôa P., Villegas I.F., Groves R.M., Benedictus R. (2019). Diagnostic of manufacturing defects in ultrasonically welded thermoplastic composite joints using ultrasonic guided waves. NDT E Int..

[6] Mohd Tahir M.F., Echtermeyer A.T. (2024). Phased Array Ultrasonic Testing on Thick Glass Fiber Reinforced Thermoplastic Composite Pipe Implementing the Classical Time-Corrected Gain Method. J. Nondestr. Eval..

[7] de Almeida P.D., Pereira G.R. (2019). Phased array inspection of glass fiber reinforced polymers pipeline joints. J. Mater. Res. Technol..

[8] Nagy P.B. (1991). Ultrasonic detection of kissing bonds at adhesive interfaces. J. Adhes. Sci. Technol..

[9] Yılmaz B., Jasiūnienė E. (2020). Advanced ultrasonic NDT for weak bond detection in composite-adhesive bonded structures. Int. J. Adhes. Adhes..

[10] Liu T., Pei C., Cheng X., Zhou H., Xiao P., Chen Z. (2018). Adhesive debonding inspection with a small EMAT in resonant mode. NDT E Int..

[11] Roth W., Giurgiutiu V. (2017). Structural health monitoring of an adhesive disbond through electromechanical impedance spectroscopy. Int. J. Adhes. Adhes..

[12] Medeiros R.d., Souza G., Marques D., Flor F., Tita V. (2021). Vibration-based structural monitoring of bi-clamped metal-composite bonded joint: Experimental and numerical analyses. J. Adhes..

[13] Dugnani R., Chang F.-K. (2017). Analytical model of lap-joint adhesive with embedded piezoelectric transducer for weak bond detection. J. Intell. Mater. Syst. Struct..

[14] Dugnani R., Zhuang Y., Kopsaftopoulos F., Chang F.-K. (2016). Adhesive bond-line degradation detection via a cross-correlation electromechanical impedance–based approach. Struct. Health Monit..

[15] Grammatikos S., Kordatos E., Matikas T., Paipetis A. (2014). Real-time debonding monitoring of composite repaired materials via electrical, acoustic, and thermographic methods. J. Mater. Eng. Perform..

[16] Martens U., Schröder K.-U. (2020). Evaluation of infrared thermography methods for analysing the damage behaviour of adhesively bonded repair solutions. Compos. Struct..

[17] Shin P.H., Webb S.C., Peters K.J. (2016). Pulsed phase thermography imaging of fatigue-loaded composite adhesively bonded joints. NDT E Int..

[18] Kryukov I., Böhm S. (2019). Prospects and limitations of eddy current shearography for non-destructive testing of adhesively bonded structural joints. J. Adhes..

[19] Kryukov I., Thiede H., Böhm S. (2017). Quality assurance for structural adhesively bonded joints by eddy current shearography. Weld World.

[20] Davis M.J., McGregor A. Assessing adhesive bond failures: Mixed-mode bond failures explained. Proceedings of the ISASI Australian Safety Seminar.

[21] Baker A.A., Wang J., Jones R., Baker A.A., Matthews N., Champagne V. (2018). Chapter Six—Adhesively Bonded Repair/Reinforcement of Metallic Airframe Components: Materials, Processes, Design and Proposed Through-Life Management.

[22] Sadeghi M.Z., Weiland J., Preisler A., Zimmermann J., Schiebahn A., Reisgen U., Schröder K.U. (2020). Damage detection in adhesively bonded single lap joints by using backface strain: Proposing a new position for backface strain gauges. Int. J. Adhes. Adhes..

[23] Sadeghi M.Z., Weiland J., Zimmermann J., Schiebahn A., Reisgen U., Schröder K.U. (2020). Experimental and FE investigations on the influential parameters in positioning and measurement of strain gauges in adhesively bonded single lap joints. Procedia Struct. Integr..

[24] Augustin T., Karsten J., Kötter B., Fiedler B. (2018). Health monitoring of scarfed CFRP joints under cyclic loading via electrical resistance measurements using carbon nanotube modified adhesive films. Compos. Part A Appl. Sci. Manuf..

[25] Dengg A., Kralovec C., Schagerl M. (2024). Damage monitoring of pinned hybrid composite-titanium joints using direct current electrical resistance measurement. Compos. Struct..

[26] Grefe H., Weiser D., Kandula M.W., Dilger K. (2020). Deformation measurement within adhesive bonds of aluminium and CFRP using advanced fibre optic sensors. Manuf. Rev..

[27] Young S., Penumadu D., Foster D., Maeser H., Balijepalli B., Reese J., Bank D., Dahl J., Blanchard P. (2020). Smart Adhesive Joint with High-Definition Fiber-Optic Sensing for Automotive Applications. Sensors.

[28] Jaiswal P.R., Kumar R.I., Saeedifar M., Saleh M., Luyckx G., De Waele W. (2021). Deformation and damage evolution of a full-scale adhesive joint between a steel bracket and a sandwich panel for naval application. Proc. Inst. Mech. Engr. Part C J. Mech. Eng. Sci..

[29] Zeng H., Yan R., Xu L., Gui S. (2020). Application study on fiber Bragg grating sensors in damage monitoring of sandwich composite joints. J. Sandw. Struct. Mater..

[30] Webb S., Shin P., Peters K., Zikry M., Stan N., Chadderdon S., Selfridge R., Schultz S. (2013). Characterization of fatigue damage in adhesively bonded lap joints through dynamic, full-spectral interrogation of fiber Bragg grating sensors: 1. Experiments. Smart Mater. Struct..

[31] Karpenko O., Khomenko A., Koricho E., Haq M., Udpa L., Chimenti D.E., Bond L.J. (2016). Monitoring of fatigue damage in composite lap-joints using guided waves and FBG sensors. Proceedings of the AIP Conference Proceedings 1706, 42nd Annual Review of Progress in Quantitative Nondestructive Evaluation.

[32] Shin C.-S., Lin T.-C. (2021). Adhesive Joint Integrity Monitoring Using the Full Spectral Response of Fiber Bragg Grating Sensors. Polymers.

[33] Shin C.-S., Lin T.-C. (2022). Hygrothermal Damage Monitoring of Composite Adhesive Joint Using the Full Spectral Response of Fiber Bragg Grating Sensors. Polymers.

[34] Andrew J.J., Arumugam V., Ramesh C. (2019). Acoustic emission characterization of local bending behavior for adhesively bonded hybrid external patch repaired glass/epoxy composite laminates. Struct. Health Monit..

[35] Shin C.S., Chiang C.C. (2005). Deformation monitoring by using optical fiber grating sensor. J. Chin. Inst. Eng..

[36] Wen S., Wang S., Chung D. (2000). Piezoresistivity in continuous carbon fiber polymer-matrix and cement-matrix composites. J. Mater. Sci..

[37] Zhang J., Zhuang R., Liu J., Mäder E., Heinrich G., Gao S. (2010). Functional interphases with multi-walled carbon nanotubes in glass fibre/epoxy composites. Carbon.

[38] Park J.-M., Kwon D.-J., Wang Z.-J., DeVries K.L. (2015). Review of self-sensing of damage and interfacial evaluation using electrical resistance measurements in nano/micro carbon materials-reinforced composites. Adv. Compos. Mater..

[39] Wang Z.-J., Kwon D.-J., Gu G.-Y., Kim H.-S., Kim D.-S., Lee C.-S., DeVries K.L., Park J.-M. (2013). Mechanical and interfacial evaluation of CNT/polypropylene composites and monitoring of damage using electrical resistance measurements. Compos. Sci. Technol..

[40] Bilotti E., Zhang H., Deng H., Zhang R., Fu Q., Peijs T. (2013). Controlling the dynamic percolation of carbon nanotube based conductive polymer composites by addition of secondary nanofillers: The effect on electrical conductivity and tuneable sensing behaviour. Compos. Sci. Technol..

[41] Hao B., Ma Q., Yang S., Mäder E., Ma P.-C. (2016). Comparative study on monitoring structural damage in fiber-reinforced polymers using glass fibers with carbon nanotubes and graphene coating. Compos. Sci. Technol..

[42] Pramanik P., Khastgir D., De S., Saha T. (1990). Pressure-sensitive electrically conductive nitrile rubber composites filled with particulate carbon black and short carbon fibre. J. Mater. Sci..

[43] Sánchez-Romate X.F., García C., Rams J., Sánchez M., Ureña A. (2021). Structural health monitoring of a CFRP structural bonded repair by using a carbon nanotube modified adhesive film. Compos. Struct..

[44] Sánchez-Romate X.F., Coca Á., Jiménez-Suárez A., Sánchez M., Ureña A. (2021). Crack sensing mechanisms of Mode-II and skin-stringer joints between dissimilar materials by using carbon nanotubes. Compos. Sci. Technol..

[45] Sánchez-Romate X.F., Sbarufatti C., Sánchez M., Bernasconi A., Scaccabarozzi D., Libonati F., Cinquemani S., Güemes A., Ureña A. (2020). Fatigue crack growth identification in bonded joints by using carbon nanotube doped adhesive films. Smart Mater. Struct..

[46] Li W., Frederick H., Palardy G. (2021). Multifunctional films for thermoplastic composite joints: Ultrasonic welding and damage detection under tension loading. Compos. Part A Appl. Sci. Manuf..

[47] Mactabi R., Rosca I.D., Hoa S.V. (2013). Monitoring the integrity of adhesive joints during fatigue loading using carbon nanotubes. Compos. Sci. Technol..

[48] Kim C.-H., Choi J.-H., Kweon J.-H. (2015). Defect detection in adhesive joints using the impedance method. Compos. Struct..

[49] Kang M.-H., Choi J.-H., Kweon J.-H. (2014). Fatigue life evaluation and crack detection of the adhesive joint with carbon nanotubes. Compos. Struct..

[50] Ladani R.B., Wu S., Zhang J., Ghorbani K., Kinloch A.J., Mouritz A.P., Wang C.H. (2017). Using carbon nanofibre sensors for in-situ detection and monitoring of disbonds in bonded composite joints. Procedia Eng..

[51] Bregar T., An D., Gharavian S., Burda M., Durazo-Cardenas I., Thakur V.K., Ayre D., Słoma M., Hardiman M., McCarthy C. (2020). Carbon nanotube embedded adhesives for real-time monitoring of adhesion failure in high performance adhesively bonded joints. Sci. Rep..

[52] Sam-Daliri O., Faller L.-M., Farahani M., Zangl H. (2021). Structural health monitoring of adhesive joints under pure mode I loading using the electrical impedance measurement. Eng. Fract. Mech..

[53] Li W., Palardy G. Mechanical and electrical properties of MWCNT/PP films and structural health monitoring of GF/PP joints. Proceedings of the SPE Automotive Composites Conference & Exhibition.

[54] Kumar S., Falzon B.G., Hawkins S.C. (2019). Ultrasensitive embedded sensor for composite joints based on a highly aligned carbon nanotube web. Carbon.

[55] Bekas D.G., Sharif-Khodaei Z., Baltzis D., Aliabadi M.F., Paipetis A.S. (2019). Quality assessment and damage detection in nanomodified adhesively-bonded composite joints using inkjet-printed interdigital sensors. Compos. Struct..

[56] Lee B.M., Loh K.J. (2015). A 2D percolation-based model for characterizing the piezoresistivity of carbon nanotube-based films. J. Mater. Sci..

[57] Li C., Chou T. (2008). Modeling of damage sensing in fiber composites using carbon nanotube networks. Comp. Sci. Technol..

[58] Hu N., Karube Y., Arai M., Watanabe T., Yan C., Li Y., Liu Y., Fukunaga H. (2010). Investigation on sensitivity of a polymer/carbon nanotube composite strain sensor. Carbon.

[59] Hu N., Karube Y., Yan C., Masuda Z., Fukunaga H. (2008). Tunneling effect in a polymer/carbon nanotube nanocomposite strain sensor. Acta Mater..

[60] Stampfer C., Helbling T., Jungen A., Hierold C. Piezoresistance of Single-Walled Carbon Nanotubes. Proceedings of the Transducers & Eurosensors ’07, The 14th International Conference on Solid-State Sensors, Actuators and Microsystems.

[61] Yang L., Anantram M.P., Han J., Lu J. (1999). Bandgap change of carbon nanotubes: Effect of small uniaxial and torsional strain. Phys. Rev. B.

[62] (2014). Standard Test Method for Lap Shear Adhesion for Fiber Reinforced Plastic (FRP) Bonding.

[63] Schneider C.A., Rasband W.S., Eliceiri K.W. (2012). NIH Image to ImageJ: 25 years of image analysis. Nat. Methods.

[64] Gonçalves J.P.M., de Moura M.F.S.F., de Castro P.M.S.T. (2002). A three-dimensional finite element model for stress analysis of adhesive joints. Int. J. Adhes. Adhes..

[65] Shin C.-S., Chen L.-W. (2023). Damage Monitoring of Composite Adhesive Joint Integrity Using Conductivity and Fiber Bragg Grating. Polymers.

[66] (2022). Standard Test Method for Tensile Properties of Plastics.

[67] (2017). Standard Test Methods for Flexural Properties of Unreinforced and Reinforced Plastics and Electrical Insulating Materials.

[68] (2021). Standard Test Method for Transition Temperatures and Enthalpies of Fusion and Crystallization of Polymers by Differential Scanning Calorimetry.

