# Supplementary material for: Evaluation of Fatigue Damage Monitoring of Single-Lap Composite Adhesive Joint Using Conductivity
Source: Polymers (Basel). 2024 Aug 22;16(16):2374. doi: 10.3390/polym16162374 (PMC11358985; doi:10.3390/polym16162374)
Supplement: Supplementary file 1 [file polymers-16-02374-s001.zip › polymers-3111673-supplementary.pdf]

Table S1 The manufacturer provided specifications of Swancor 2261-A/BS epoxy resin (Swancor Industrial Corp., Nantou, Taiwan) .

epoxy resin	SW 2261-A/BS	Test methods
Tensile Strength （MPa）	75~95	ASTM D638 [66]
Tensile modulus （MPa）	2,800~3,700	ASTM D638 [66]
Elongation at break （%）	>5.0	ASTM D638 [66]
Bending strength （MPa）	110~140	ASTM D790 [67]
Bending modulus（MPa）	2,800~3,700	ASTM D790 [67]
T_g （˚C）	110~130	ASTM D3418 [68]

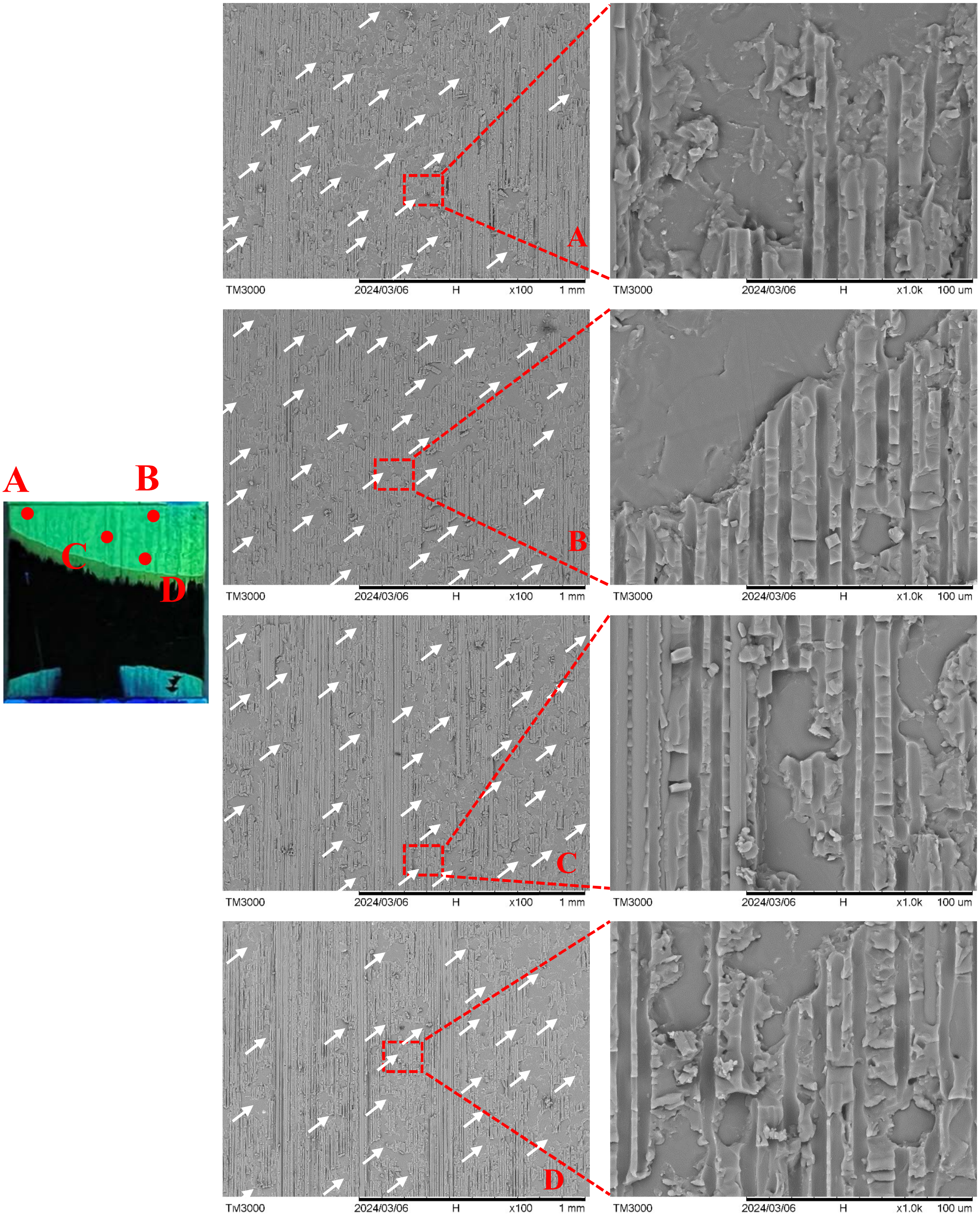

Figure S1. More microscopic and magnified views of the dotted rectangle joint fracture views of specimen *P*; extensive white arrows show interfacial debonding sites.

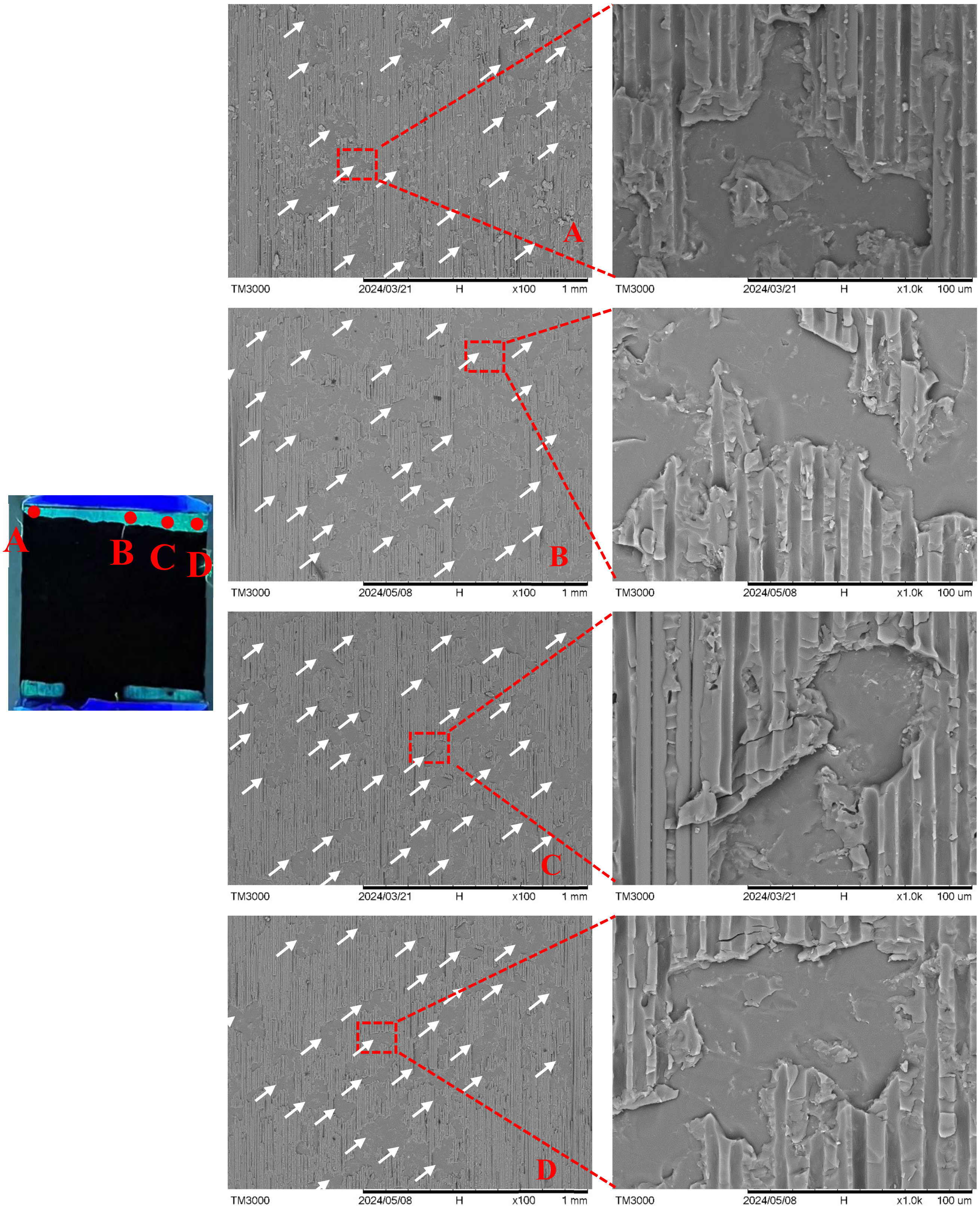

Figure S2. More microscopic and magnified views of the dotted rectangle joint fracture views of specimen *Q*; extensive white arrows show interfacial debonding sites.

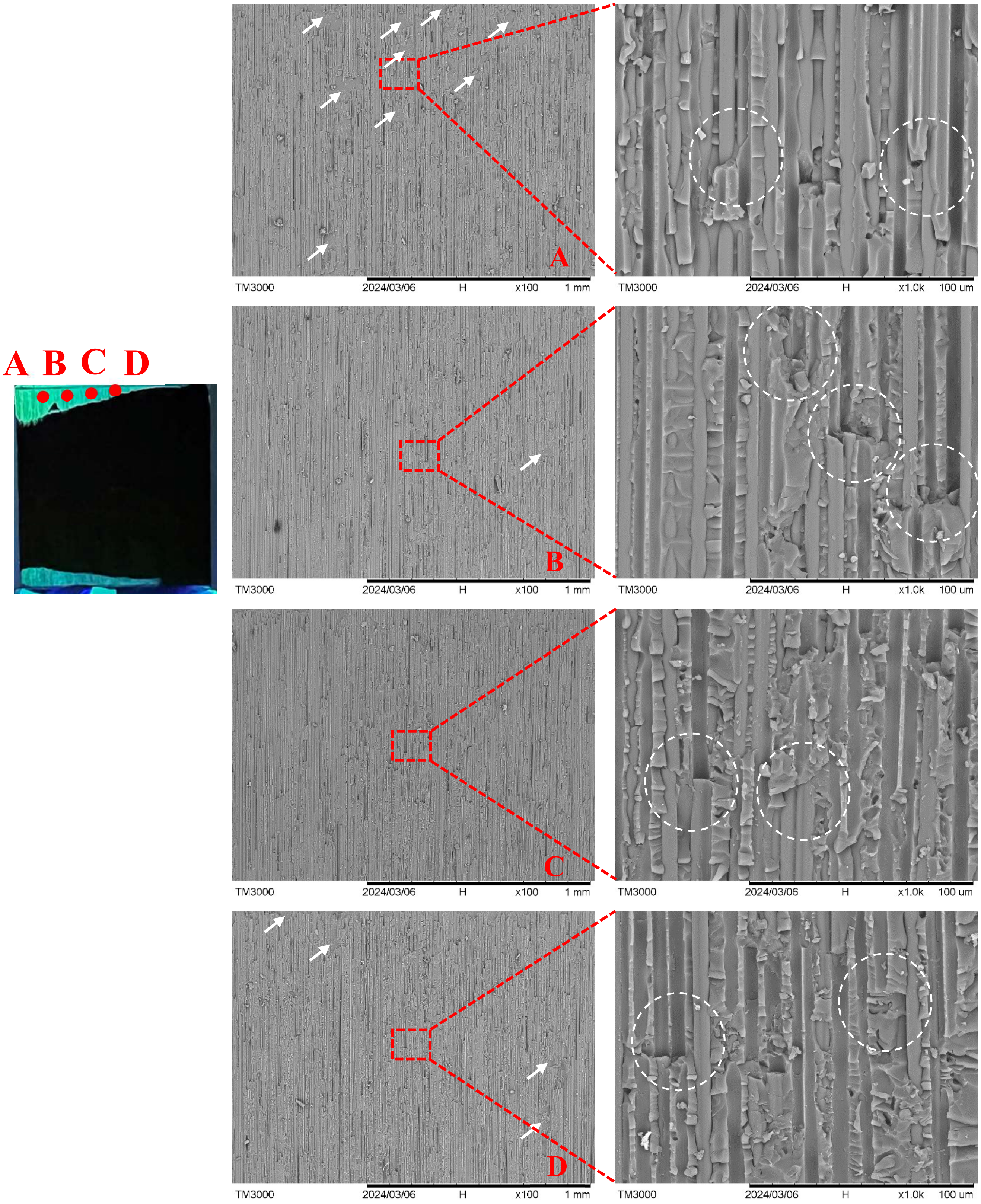

Figure S3. More microscopic and magnified views of the dotted rectangle joint fracture views of specimen *R*; sparing white arrows show interfacial debonding sites and broken circles indicate intra- or interlaminar debonding.

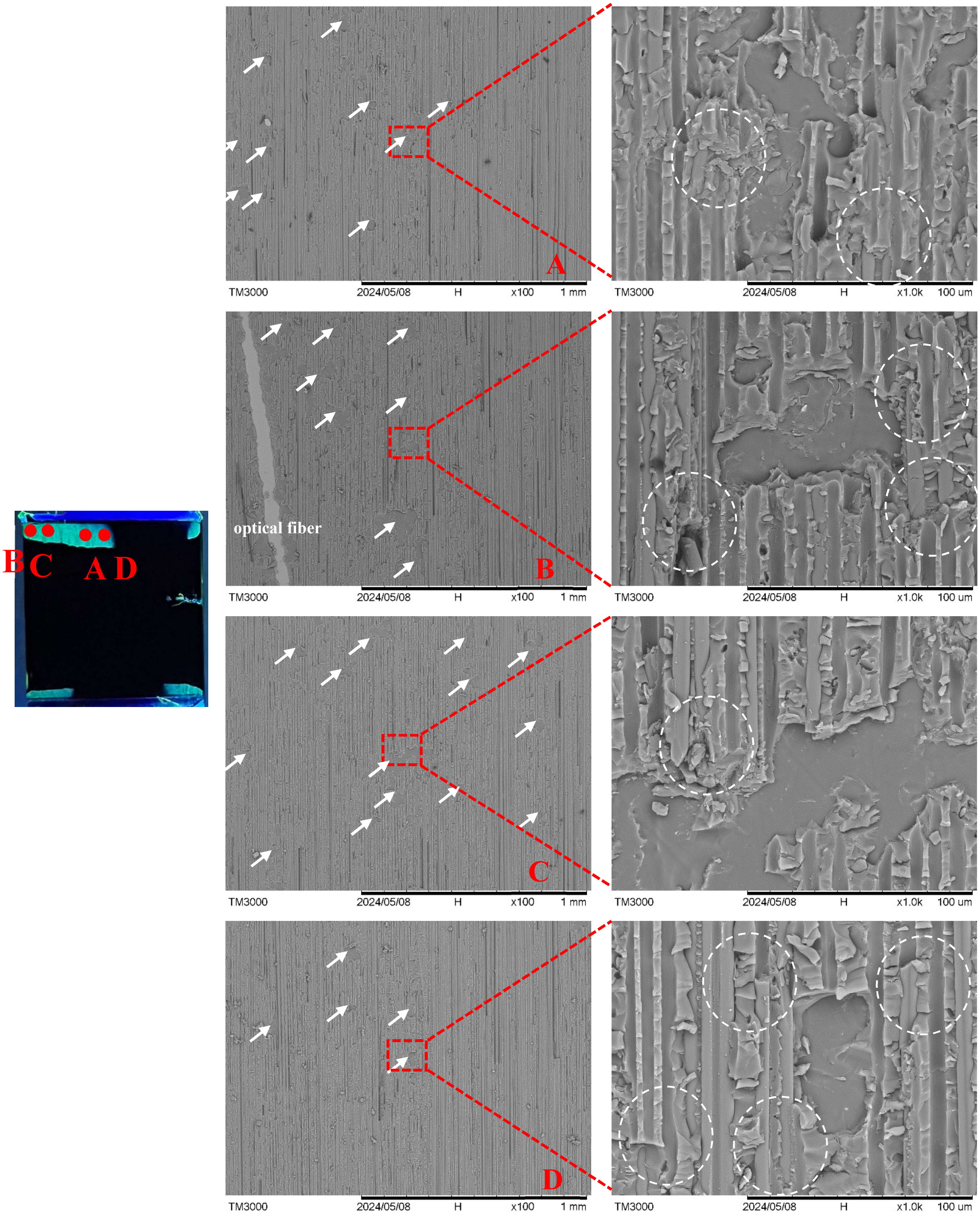

Figure S4. More microscopic and magnified views of the dotted rectangle joint fracture views of specimen *S*; sparing white arrows show interfacial debonding sites and broken circles indicate intra- or interlaminar debonding.

References

66. ASTM D638-22; Standard Test Method for Tensile Properties of Plastics, ASTM International, West Conshohocken, PA, USA, 2022.
67. ASTM D790-17; Standard Test Methods for Flexural Properties of Unreinforced and Reinforced Plastics and Electrical Insulating Materials, ASTM International, West Conshohocken, PA, USA, 2017.
68. ASTM D3418-21; Standard Test Method for Transition Temperatures and Enthalpies of Fusion and Crystallization of Polymers by Differential Scanning Calorimetry, ASTM International, West Conshohocken, PA, USA, 2021.
